# Induction of FoxP3 Pre-mRNA Alternative Splicing to Enhance the Suppressive Activity of Regulatory T Cells from Amyotrophic Lateral Sclerosis Patients

**DOI:** 10.3390/biomedicines12051022

**Published:** 2024-05-07

**Authors:** Dmitry D. Zhdanov, Yulia A. Gladilina, Varvara G. Blinova, Anna A. Abramova, Anastasia N. Shishparenok, Daria D. Eliseeva

**Affiliations:** 1 Laboratory of Medical Biotechnology, Institute of Biomedical Chemistry, Pogodinskaya St. 10/8, 119121 Moscow, Russia; gladilinaya@ibmc.msk.ru (Y.A.G.); varya.blinova@list.ru (V.G.B.); abramova.neurology@gmail.com (A.A.A.); a.shishparyonok@ibmc.msk.ru (A.N.S.); 2 Department of Biochemistry, Peoples’ Friendship University of Russia named after Patrice Lumumba (RUDN University), Miklukho-Maklaya St. 6, 117198 Moscow, Russia; 3 Research Center of Neurology, Volokolamskoe Shosse, 80, 125367 Moscow, Russia; ddeliseeva@gmail.com

**Keywords:** regulatory T cells, alternative splicing, FoxP3, suppressive activity, splicing-switching oligonucleotides, amyotrophic lateral sclerosis

## Abstract

Forkhead box protein 3 (FoxP3) is a key transcription factor responsible for the development, maturation, and function of regulatory T cells (Tregs). The FoxP3 pre-mRNA is subject to alternative splicing, resulting in the translation of multiple splice variants. We have shown that Tregs from patients with amyotrophic lateral sclerosis (ALS) have reduced expression of full-length (FL) FoxP3, while other truncated splice variants are expressed predominantly. A correlation was observed between the reduced number of Tregs in the peripheral blood of ALS patients, reduced total FoxP3 mRNA, and reduced mRNA of its FL splice variant. Induction of FL FoxP3 was achieved using splice-switching oligonucleotides capable of base pairing with FoxP3 pre-mRNA and selectively modulating the inclusion of exons 2 and 7 in the mature mRNA. Selective expression of FL FoxP3 resulted in the induction of CD127^low^, CD152, and Helios-positive cells, while the cell markers CD4 and CD25 were not altered. Such Tregs had an increased proliferative activity and a higher frequency of cell divisions per day. The increased suppressive activity of Tregs with the induced FL FoxP3 splice variant was associated with the increased synthesis of the pro-apoptotic granzymes A and B, and perforin, IL-10, and IL-35, which are responsible for contact-independent suppression, and with the increased ability to suppress telomerase in target cells. The upregulation of Treg suppressive and proliferative activity using splice-switching oligonucleotides to induce the predominant expression of the FoxP3 FL variant is a promising approach for regenerative cell therapy in Treg-associated diseases.

## 1. Introduction

The onset, the development, and the progression of some autoimmune diseases are associated with a decrease in the number of regulatory T cells (Tregs) in the peripheral blood [1,2,3,4]. Along with reduction in the number, the ability of Tregs to suppress autoreactive target lymphocytes can also be affected in autoimmune patients [5,6,7,8,9]. To date, the reasons and exact molecular mechanisms of such abnormalities remain to be clarified.

Amyotrophic lateral sclerosis (ALS) is a devastating neurodegenerative disorder characterized by the progressive loss of motor neurons in the brain and spinal cord, resulting in severe functional impairment. It can present with both motor and non-motor symptoms, and the clinical phenotypes of the disease can vary [10]. ALS typically begins with focal weakness that spreads to other skeletal muscles, including the diaphragm [11]. Neuroinflammation is suggested to be another key feature of ALS pathogenesis, along with activated microglia and astroglia in the central nervous system and proinflammatory peripheral lymphocytes and macrophages. Although various mutations can lead to accumulation of toxic proteins in the motor neurons, the pro-inflammatory cascade that occurs along with disease progression shows many common features [12].

The role of Tregs in ALS has been studied extensively. These cells suppress the pro-inflammatory action of Th17-cells and macrophages. In patients with ALS, lower numbers of Tregs are associated with increased mortality rate and a shorter lifespan. The function of Tregs, specifically their ability to suppress the proliferation of responder T cells, is severely impaired. This suggests that the overall impact of Tregs on survival outcome is predominantly caused by a loss of function rather than a reduction in number [13,14,15,16]. Another study has demonstrated that a high frequency of CD4^+^FOXP3^−^ effector T cells in blood and cerebrospinal fluid at the moment of ALS diagnosis is associated with worse survival chances. By contrast, a high frequency of activated Tregs cells and a high ratio between activated and resting Treg cells in peripheral blood are associated with a better survival prognosis [17].

It was shown in many studies that the ability of Tregs to control the immune response to autoantigens is under the control of the transcription factor Forkhead Box Protein 3 (FoxP3) [18,19,20,21]. Due to its ability to induce the transcriptional activity of many genes, FoxP3 determines the suppressor activity of Tregs, as well as their differentiation and proliferative intensities [22,23]. Pre-mRNA transcribed from the *FoxP3* gene is subjected to alternative splicing, leading to the selective inclusion or exclusion of exon 2 and/or exon 7 in the mature mRNA. This process results in the formation of four alternative splice variants. The full-length (FL) mRNA encodes the longest variant that contains all the exons, including exons 2 and 7. Two variants are truncated and encode the proteins with the deletions of either of each exon 2 (∆2 splice variant) or exon 7 (∆7 splice variant) coding regions. The shortest variant encodes the smallest protein with the deletions of both exons 2 and 7 (∆2∆7 splice variant) [24,25].

A number of studies have been performed to clarify the effect of FoxP3 splice variants on the function of Tregs and their association with the autoimmune diseases [24,26,27,28,29]. The conclusion from these studies is that the FL FoxP3 splice variant determines the normal proliferation and functional activity of Tregs, while truncated variants have a negative effect on these crucial cellular processes.

In this work, we aimed to investigate the expression of FoxP3 splice variants in peripheral blood Tregs from ALS patients. We also aimed to create Tregs selectively expressing only FL FoxP3 and monitor their suppressive activity and proliferation.

## 2. Materials and Methods

### 2.1. Demographic Characteristics of Study Participants

The group of 20 patients with definite ALS was recruited by the outpatient department of the Research Center of Neurology. Only patients aged 18 and older were recruited. All procedures with the patients were performed in accordance with the Helsinki Declaration of 1964 and its later updates and with the ethical standards of the National Research Committee. The study, Number 12-3 of 28 December 2020, was approved by the Local Ethics Committee of the Scientific Center for Neurology. ALS was classified as spinal onset or bulbar onset ALS depending on the anatomical area first affected. Clinical staging was performed according to King’s staging system [30]. ALS patients were clinically evaluated using the Revised Amyotrophic Lateral Sclerosis Functional Rating Scale (ALSFRS-R) [31]. Active smokers, patients with a history of malignancies, diabetes mellitus, or cardiovascular disease, and patients with acute and chronic infectious diseases were excluded from the study.

The group of healthy donors consisted of 20 volunteers of age- and sex-matched to the ALS group. Healthy donors were subjected to neurological assessment and physical examination to exclude any neurological disease. The main demographic characteristics of the study groups are shown in Table 1.

All ALS patients were diagnosed with definite ALS according to the El Escorial criteria (2000) [32]. Disease duration was measured as the time of first symptom onset to study enrollment. The clinical data are shown in Table 2.

The mean disease duration in the ALS group was 22.0 ± 17.8 months, ranging from 4 to 67 months. None of the patients had a history of familial ALS, so their disease could be classified as sporadic. Eighteen patients (95%) had spinal-onset ALS, while three patients (15%) had bulbar-onset ALS. None of the patients had any cognitive or behavioral symptoms indicative of frontotemporal dementia.

### 2.2. Blood Sampling and Treg Purification

Written informed consent to participate in the study was obtained from the patients and donors before blood sampling. Blood samples were obtained via venipuncture into vacuum tubes with K_2_EDTA anticoagulant. Density gradient centrifugation using 1.077 g/mL Ficoll (Paneco, Moscow, Russia) was used to obtain peripheral blood mononuclear cells. Then, Tregs were isolated from peripheral blood mononuclear cells by immune-magnetic separation using the CD4+CD25+Treg isolation kit (Miltenyi Biotec, Bergisch Gladbach, Germany).

### 2.3. Flow Cytometry of Treg-Associated Cell Markers

The catalog numbers for key antibodies, antibody cocktails, and reagents used in flow cytometry are presented in Table S1 in the Supplementary Materials. The gaiting strategies for flow cytometry were set up according to the manufacturer’s protocol. A MACS Quant Analyzer 10 flow cytometer and associated MACSQuantify 3.0 Software (all from Miltenyi Biotec, Bergisch Gladbach, Germany) were used for the flow cytometry study. The percentage of Tregs in peripheral blood samples and the purity of isolated cells were determined using the Treg Surface Marker Analysis Cocktail (CD45-VioBlue, CD4-FITC, CD25-APC, CD127-PE, Miltenyi Biotec, Bergisch Gladbach, Germany). The percentage of cells with Treg-associated markers was measured after the labeling of cells with anti-CD4–FITC, anti-CD25–APC, anti-CD127–FITC, anti-CD152–APC, or anti-CD39–FITC (all from Miltenyi Biotec, Bergisch Gladbach, Germany). To detect the transcription factor Helios, cells were fixed and permeabilized using the Transcription Factor Buffer Set (BD Pharmingen, East Rutherford, NJ, USA) and then labeled with anti-Helios-PE (Miltenyi Biotec, Bergisch Gladbach, Germany). To study the cells’ ability to produce granzymes A and B and perforin, the cells were stimulated with the Stimulation Cocktail + Protein Transport Inhibitor (eBioscience Inc., San Diego, CA, USA) followed by labeling with anti-granzyme A-PE, anti-granzyme B-PE, or anti-perforin-FITC (all from Miltenyi Biotec, Bergisch Gladbach, Germany).

Mean fluorescence intensity (MFI) was used to measure the intensity of cell marker expression.

### 2.4. Treg Transfection and Cultivation

In our previous work [29], we demonstrated that selective modulation of exon 2 and 7 deletion or insertion in mature RNA can be achieved by using specific SSOs. In this work, to induce the expression of FL FoxP3, cells were transfected with 36-mer SSOs capable of selectively including exon 2 (#Ins2, 5′-ATGTGGCCTGTCCAGGAGGAGTGCCTGTAAGTGGGG-3′) and exon 7 (#Ins7, 5′-GGCACTCACGTTCTCCTTCTCCAGCACCAGCTGTGA-3′) into mature FoxP3 mRNA. The SSOs were custom synthesized by Evrogen (Moscow, Russia), uniformly modified with 2′-O-(2-methoxy) ethyl sugars, had a phosphorothioate backbone, and carried a 5′-methyl cytosine to avoid intracellular degradation [33,34]. SSOs were conjugated with the fluorescent dyes #Ins2-Cy5.5 and #Ins7-Cy3 at the 5′ ends. Non-specific oligonucleotides (#Con1, 5′-ATGTGCCGTAGGTGAGGCCTCACGTTCGTTAAACGG-3′ and #Con2, 5′-GTGAGGCCTCACGTTCGTTAAACGGATGTGCCGTAG-3′) of the same size and chemical modifications were used as a control. Lipofectamine 2000 (Invitrogen, Grand Island, NY, USA) was used for transfection according to the manufacturer’s protocol.

Transfected cells were cultured ex vivo within 96 h in Treg culture media (RPMI-1640 (Paneco, Moscow, Russia), 10% fetal bovine serum (Capricorn Scientific, Ebsdorfergrund, Germany), 5 µg/mL anti-CD3 monoclonal antibodies (MedBioSpectr, Moscow, Russia), 2 µg/mL anti-CD28 antibodies (eBiosciences, San Diego, CA, USA), and 100 U/mL of rHu IL-2 (R&D Systems, Minneapolis, MN, USA), as we have previously described [25,35].

Cell imaging was performed by the inverted microscope Biomed 3I (Biomed, Saint Petersburg, Russia). The total number of cells during the cultivation time was measured daily using a Cell Viability Analyzer Vi-Cell XR (Beckman Coulter, Brea, CA, USA) via the Trypan Blue exclusion test. The equation used was *N_d_* = *N*_0_2*^df^* (*N_d_* is the number of cells on a given day (*d*); *N*_0_ is the initial number of cells on day 0 of cultivation; *f* is the frequency of cell cycles per day [34,36]).

The proportion of proliferating transfected Tregs was measured daily by flow cytometry after cell labeling with vital dye from the CellTrace Violet Cell Proliferating Kit (Life Technologies, Carlsbad, CA, USA).

### 2.5. Quantitative PCR (qPCR) and Western Blotting

Quantitative PCR (qPCR) was utilized to measure the proportion of FoxP3 mRNA splice variants. The protocols for isolating RNA, reverse transcription, primers used, amplification cycles, hardware, and software are described in detail in our previous works [29,37]. The average RNA levels of three genes (beta-actin, 18S, and glyceraldehyde-3-phosphate dehydrogenase (GAPDH)) were utilized as a reference to normalize the determined mRNA levels of the FoxP3 splice variants.

The detection of the protein splice variant with exon 2 was performed by Western blotting using exon-specific FoxP3 antibodies. Clone 150D can recognize exon 2, while clone 259D (both from BioLegend, San Diego, CA, USA) is specific to the epitope located after exon 2 which is common for all splice variants. The Western blotting protocol was previously described by us [38]. GAPDH was used as a loading control. The catalog numbers for antibodies used for Western blotting are presented in Table S1 in the Supplementary Materials.

### 2.6. Suppression Assays and the Detection of Suppressive Cytokines

Mixed lymphocyte reaction was performed to study the suppressive activity of Tregs in a contact-dependent manner. The protocol that we previously described [39] was used. Tregs, at 96 h post-transfection, were co-incubated for five days with autologous target CD4^+^CD25^−^ T lymphocytes labeled with the vital dye carboxyfluorescein succinimidyl ester (CFSE, Life Technologies, Carlsbad, CA, USA). Allogeneic peripheral blood mononuclear cells treated with mitomycin C (Kyowa Hakko Kogyo, Tokyo, Japan) were also used. The proportions of Tregs to target cells were within the range of 1:2 to 1:96. The proliferation of target cells was determined by the number of cells with reduced CFSE intensity detected by flow cytometry.

The telomeric repeat amplification protocol (TRAP) [40,41] was performed to test the ability of Tregs to inhibit telomerase in target cells. Briefly, Tregs at 96 h post-transfection were co-incubated with autologous target CD4^+^CD25^−^ T lymphocytes in a contact-independent manner using 8 mm membrane inserts (Millicell Culture Inserts, Millipore, Bedford, MA, USA) for 12 h as we have described previously [42,43]. Target cells were subjected to TRAP and the results of amplification were visualized by the electrophoresis in a 12% polyacrylamide gel stained with SYBR Green I. Telomerase activity in Jurkat cells (T lymphoblast leukemia cells, ATCC, Manassas, VA, USA) was used as the reference activity.

The concentrations of the Treg-associated suppressive cytokines IL-10 and IL-35 were detected in growth media in 96 h post-transfection using the 12-Plex Bio-Plex Pro™ Human Treg Cytokine Panel (Bio-Rad, Hercules, CA, USA) according to manufacturer’s protocol. The immunoanalyzer Bio-Plex 200 System (Bio-Rad, Hercules, CA, USA) was used, and the results were subsequently processed by the Bio-Plex Manager 6.0 Properties application (Bio-Rad, Hercules, CA, USA).

### 2.7. Statistics

Statistical differences were analyzed by the Mann–Whitney U test using SPSS 25 software (IBM SPSS Statistics, Armonk, NY, USA). *p* ≤ 0.05 was considered significant and marked with *. GraphPad Prism 8.0 (GraphPad Software, Inc., New York, NY, USA) was used to construct the graphs.

## 3. Results

### 3.1. FoxP3 Full-Length Splice Variant Expression Is Reduced in Tregs from ALS Patients

**Figure 1:**
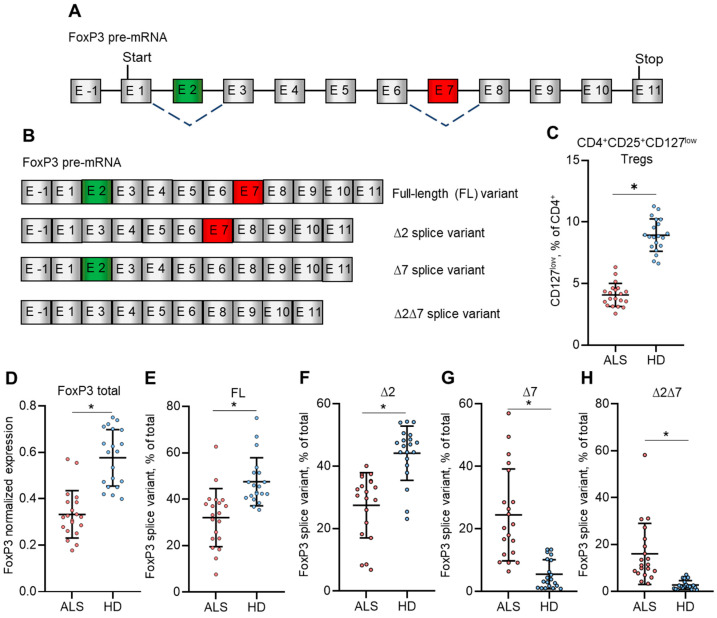
Reduced proportion of Tregs associates with reduced expression of total FoxP3 and the FL FoxP3 splice variant. (**A**) Schematic presentation of FoxP3 pre-mRNA. It consists of one non-coding exon E-1 and 11 protein coding exons E1–E11. The locations of start codon and stop codon are shown. Exon 2 (green box) and exon 7 (red box) are subjected to alternative splicing (shown as a dotted line). (**B**) Schematical presentation of FoxP3 mature splice variants. The full-length (FL) variant has all 11 exons. The variant ∆2 lacks exon 2. The variant ∆7 lacks exon 7. The variant ∆2∆7 lacks both exons 2 and 7. (**C**) The proportion of Tregs in the peripheral blood of amyotrophic lateral sclerosis (ALS) patients and healthy donors (HD). (**D**) The expression of total FoxP3 mRNA among ALS patients and healthy donors was determined by qPCR. The expression of FoxP3 splice variants (**E**) FL, (**F**) ∆2, (**G**) ∆7, and (**H**) ∆2∆7 among ALS patients and healthy donors determined by qPCR. Individual values for each patient or healthy donor are shown. The results are presented as the mean ± standard deviation. *n* = 20 individuals in ALS or the group of healthy donors. * *p* ≤ 0.05 by Mann–Whitney U test.

The percentage of CD4^+^CD25^+^CD127^low^ Tregs was measured in the peripheral blood of ALS patients and healthy donors by flow cytometry. We found a significant decrease in Tregs in ALS patients (median 4.0%; mean 8.9 ± 0.9%) in comparison to Tregs from healthy donors (median 8.8%; mean 8.9 ± 1.3%) (Figure 1C). The decrease in Tregs in peripheral blood corresponded with the decreased expression of total FoxP3 mRNA (Figure 1D) and with the changes in its splice variants (Figure 1E–H). The normalized mRNA level of total FoxP3 measured by qPCR was 0.333 ± 0.102 (median 0.321) in ALS patients and 0.577 ± 0.121 (median 0.603) in healthy donors. The proportion of the FL FoxP3 splice variant was decreased to 32.1 ± 4.7% (median 33.6%) in Tregs from ALS patients compared to 48.1 ± 3.5% (median 43.9%) in healthy donors. The proportion of the ∆2 splice variant was also decreased in ALS patients (27.5 ± 4.4%, median 31.2%) compared to the group of healthy donors (45.1 ± 3.9%, median 47.1%). However, the proportions of splice variants with the deletion of exon 7 were induced in the ALS group: the ∆7 splice variant was 24.5 ± 5.2% (median 20.9%) in the ALS group vs. 5.6 ± 1.3% (median 3.3%) in the group of healthy donors, and the ∆2∆7 splice variant 16.0 ± 3.7%, (median 11.3%) in the ALS group vs. (3.0 ± 0.8%, median 2.3%) in the group of healthy donors. Thus, the results of this study demonstrated the correspondence between decreased numbers of Tregs in the peripheral blood of ALS patients, decreased total FoxP3 mRNA, and the decreased mRNA of its FL splice variant. The individual proportions of FoxP3 splice variants in Tregs isolated from peripheral blood from patients with ALS or healthy donors are presented in Figure S1 in the Supplementary Materials.

### 3.2. The Induction of FL FoxP3 Splice Variant with Specific SSOs

**Figure 2:**
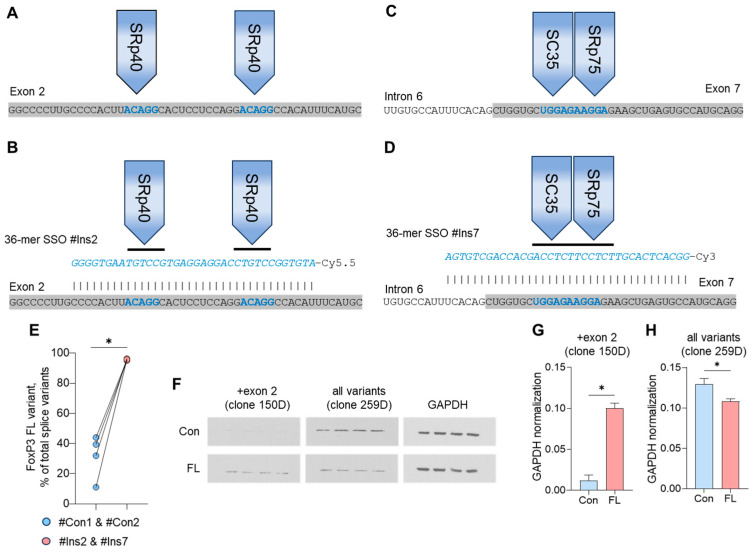
Induction of full-length (FL) FoxP3 in Tregs from ALS patients using splice-switching oligonucleotides (SSOs). Schematic presentation of FoxP3 pre-mRNA sequences with the sites (cis-elements) of interaction with splicing regulatory (SR) proteins. Exons are shown on a gray background. Introns are shown on a white background. Cis-elements are shown in a bold blue font. (**A**) Two cis-elements ACAGG are binding sites for SR protein SRp40 located in exon 2, leading to the deletion of exon 2 in mature RNA. (**B**) Base-pairing of the #Ins2 36-mer SSO (shown in blue italics) to FoxP3 pre-mRNA blocks the binding of SR proteins to their binding sites, resulting in exon 2 retention. (**C**) Two cis-elements UGGAG and AAGGA are binding sites for SC35 and SRp75 SR proteins, located in exon 7, leading to the deletion of exon 7 in mature RNA. (**D**) The base-pairing of the #Ins7 36-mer SSO to FoxP3 pre-mRNA blocks the binding of SR proteins to their binding sites resulting in exon 7 retention. (**E**) The qPCR results demonstrate the induction of the full-length (FL) FoxP3 splice variant after transfection of Tregs with the #Ins2 & #Ins7 SSOs. Individual values for control cells (Con) and those with induced FL FoxP3 are shown. (**F**) The Western blotting results show the induction of the FL splice variant after transfection with the #Ins2 & #Ins7 SSOs. Clone 150D is specific to exon 2, while clone 259D is specific to the epitope after exon 2, which is common for all FoxP3 splice variants. (**G**,**H**) Quantified by densitometry and normalized by GAPDH results of Western blotting. The results are shown as the mean ± standard deviation. *n* = 4. * *p* ≤ 0.05 by Mann–Whitney U test.

Tregs from ALS patients were transfected with SSOs that could selectively induce the insertion of exons 2 and 7 (i.e., #Ins2 and #Ins7 oligonucleotides) or control nonspecific oligonucleotides #Con1 and #Con2 and subjected to qPCR 96 h after transfection. Base-pairing of FoxP3 pre-mRNA with 36-mer SSOs in the regions of exon 2 (Figure 2A,B) or exon 7 (Figure 2C,D) sterically blocks the binding of splicing regulatory proteins to their binding sites. The transfection efficiency was monitored daily and was not lower than 76–74% in four days after transfection (Figure S2 in the Supplementary Materials). Treg transfection with the SSOs #Ins2 and #Ins7 could induce the predominant expression of FL FoxP3 up to 95.1–96.4% (Figure 2E). In Tregs transfected with #Con1 and #Con2, the proportion of FL FoxP3 was 44.1–11.1% which was not different from initial non-transfected cells from the same patients. The mRNA expression of FoxP3 splice variants in transfected Tregs is shown in Table S2 in the Supplementary Materials.

The results of FL FoxP3 induction obtained by qPCR were confirmed by Western blotting. Using antibody clone 150D (specific for the epitope encoding exon 2), it was shown that control cells had a very low level of +exon 2 FoxP3 protein, which became more than 5 times more abundant after induction of the FL variant (Figure 2A,G). Antibody clone 259D, which is specific for the epitope located right after exon 2 and common to all splice variants, was used to detect the FoxP3 protein in both the control cells and the cells with FL FoxP3 (Figure 2A,H).

The results of this experiment demonstrated that the transfection of Tregs with #Ins2 and #Ins7 oligonucleotides allowed us to obtain cells with selective expression of the FL FoxP3 splice variant.

### 3.3. Immunophenotype of Tregs with FL FoxP3

**Figure 3:**
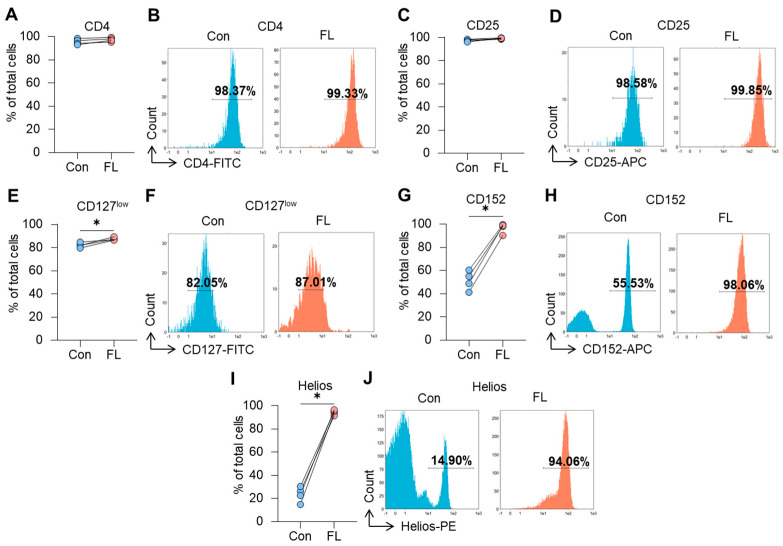
Immunophenotype of Tregs expressing the full-length (FL) FoxP3 splice variant. The results of flow cytometry study of control (Con) Tregs and Tregs with full-length (FL) FoxP3 for (**A**) CD4, (**C**) CD25, (**E**) CD127^low^, (**G**) CD152, and (**I**) Helios. Individual values for control cells and those with induced FL FoxP3 are shown. (**B**,**D**,**F**,**H**,**J**) Representative flow cytometry plots for control Tregs and with FL FoxP3. *n* = 4. * *p* ≤ 0.05 vs. control cells by Mann–Whitney U test.

Flow cytometry demonstrated that Tregs expressing FL FoxP3 were all CD4^+^-positive (95.2–99.3%) and did not differ from control cells (93.0–98.4%) (Figure 3A,B). The MFI values of cell markers for transfected Tregs are presented in Table 3.

The number of cells with a CD25^+^ phenotype also did not change between FL FoxP3 Tregs (98.6–99.8%) and control Tregs (96.3–98.6%) (Figure 3C,D). A slight but significant induction of cells with the CD127^low^ phenotype was observed in Tregs after FL FoxP3 induction (86.4–89.3%) compared to control cells (79.6–84.8%) (Figure 3E,F).

The induction of FoxP3 FL splice variant led to a strong induction of cells with the receptor CD152 (90.2–99.6%) (Figure 3G,H) and the transcription factor Helios (91.4–96.6%) (Figure 3I,J). In control Tregs, these numbers comprised 41.3–60.4% for CD152 and 14.9–30.3% for Helios.

The results of this study demonstrated that the selective expression of FL FoxP3 resulted in the induction of CD127^low^, CD152, and Helios-positive cells while the cell markers CD4 and CD25 were not changed.

### 3.4. Proliferative Activity of Tregs with Selective Expression of FL FoxP3 Splice Variant

**Figure 4:**
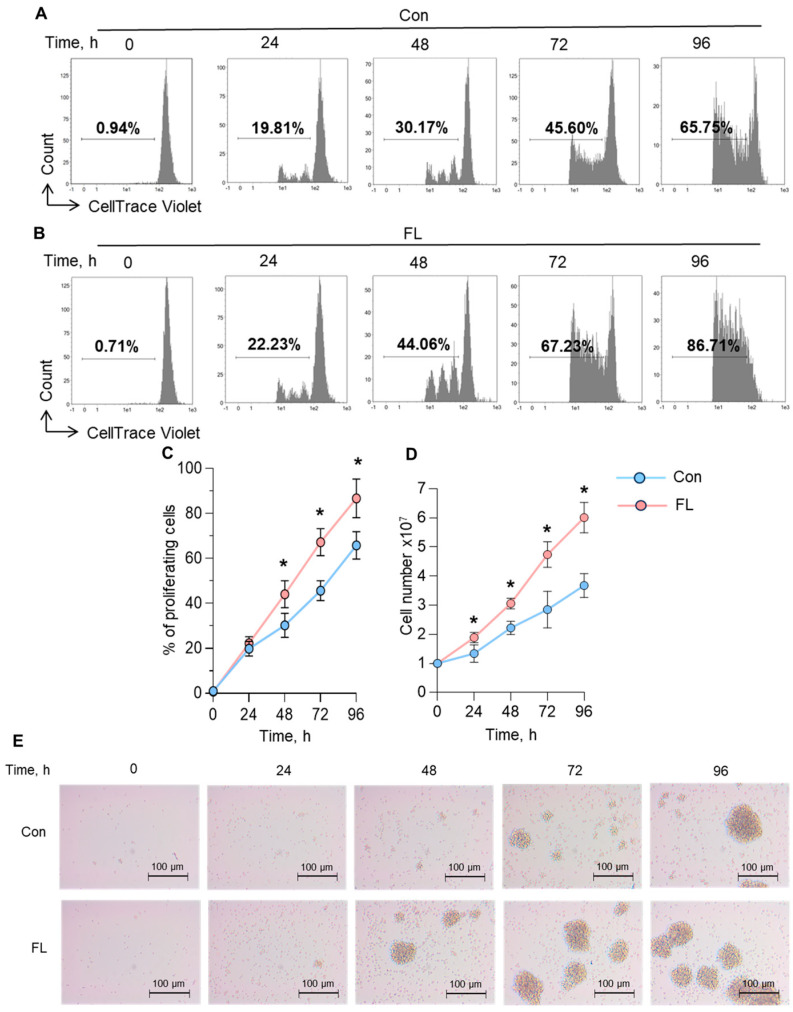
Increased proliferative capacity of Tregs with the induced full-length (FL) FoxP3 splice variant. Representative flow cytometry plots demonstrate the proliferation of (**A**) control cells (Con) and (**B**) Tregs with FL FoxP3. The decrease in CFSE intensity is associated with increased proliferation. (**C**) Percentage of proliferating cells determined by flow cytometry. (**D**) Total cell number during 96 h of proliferation. (**E**) Bright field microscopy for cells proliferating within 96 h. The results are shown as the mean ± standard deviation. *n* = 4. * *p* ≤ 0.05 vs. control cells by Mann–Whitney U test.

To monitor the proliferative activity of Tregs with FL FoxP3, the frequency of cell division was monitored every 24 h by counting cells with a reduced CellTrace Violet signal by flow cytometry. Tregs with the induced FL FoxP3 splice variant demonstrated a significantly higher proportion of proliferating cells within the cultivation time (Figure 4A–C): at 96 h post-transfection, 86.1 ± 8.6% of Tregs with FL FoxP3 underwent a round of division compared to 65.8 ± 6.3% of control Tregs. The frequency of division may not correspond to the proliferation rate because some cells may not be able to divide under cell culture conditions. We counted the total number of cells within 96 h of culturing after transfection. Full-length FoxP3 expressing cells proliferated at a higher rate, and at 96 h post-transfection, their total number reached 6.01 ± 0.52 × 10^7^ compared to 3.68 ± 0.41 × 10^7^ of control cells (Figure 4D). The frequency of cell division per day was calculated based on the daily number of cells. The frequency was 0.65 for FL FoxP3 expressing Tregs and 0.56 for control Tregs. The Tregs were photographed using an inverted microscope during the time of proliferation (Figure 4E) to monitor their morphology. Control Tregs started to form cell aggregates or so-called proliferation clusters in 72 h post-transfection, while FL FoxP3 cells were able to form them in 48 h and the number of clusters was higher. The size of proliferation clusters was 60–100 µm and did not differ between the Treg groups.

The results of this study demonstrated that Tregs with selective expression of FL FoxP3 have an increased proliferative activity.

### 3.5. Suppressive Activity of Tregs with Selective Expression of FL FoxP3 Splice Variant

**Figure 5:**
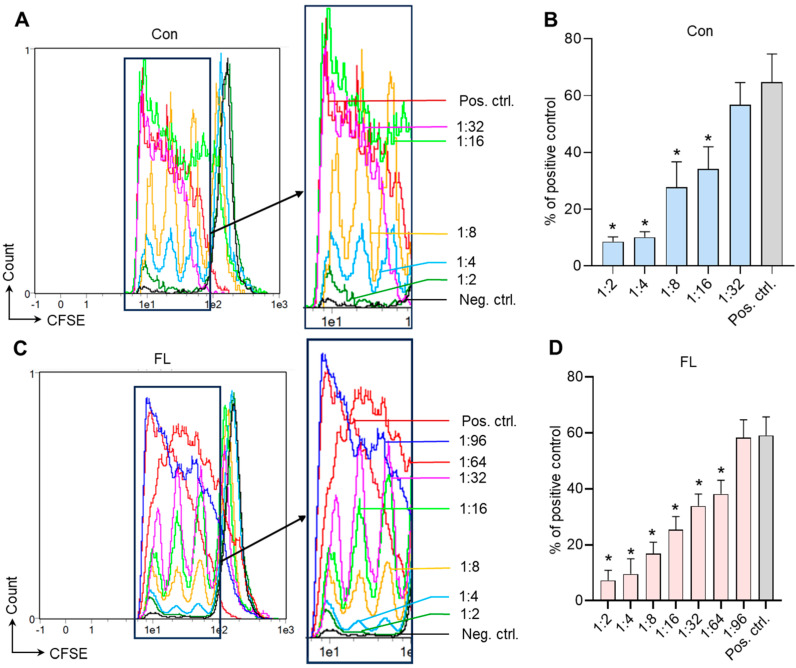
Tregs with full-length (FL) FoxP3 demonstrate increased suppressive activity. Tregs selectively expressing the FL splice variant or control transfected Tregs were subjected to a mixed lymphocyte reaction to study their suppressive activity. Proliferative plots for target responder CD4^+^CD25^+^ T-cells co-incubated with (**A**) control Tregs or (**C**) Tregs with FL FoxP3 at different ratios. (**B**,**D**) The results of the MLR assay. Con.—control Tregs. FL—Tregs with FL FoxP3. Neg. ctrl.—negative control (target cells only). Pos. ctrl.—positive control (target cells co-incubated with mitomycin-C-treated stimulating cells). The results are shown as the mean ± standard deviation. N = 4. * *p* ≤ 0.05 vs. Pos. ctrl. by Mann–Whitney U test.

An MLR assay was performed to test the suppressive activity of Tregs with FL FoxP3. Control Tregs were able to suppress the proliferation of target responder CD4^+^CD25^−^ T cells at the ratio 1:16 (Figure 5A,B), while FL FoxP3 Tregs were four times more active and could suppress the target cells even at the ratio 1:64 (Figure 5C,D).

**Figure 6:**
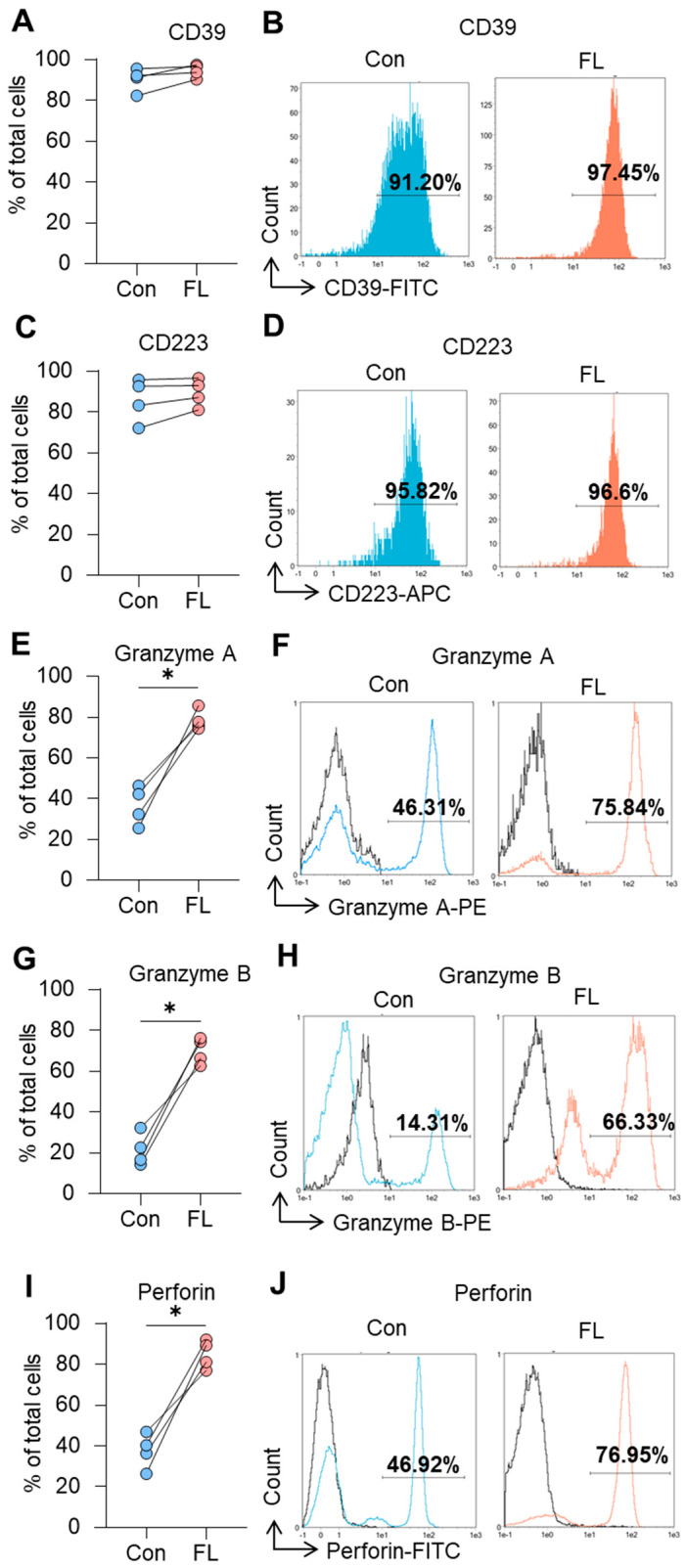
Increased expression of the molecules associated with the contact-dependent suppressive function of Tregs. The results of a flow cytometry study of control (Con) Tregs and Tregs with full-length (FL) FoxP3 for (**A**) CD39, (**C**) CD223, (**E**) granzyme A, (**G**) granzyme B, and (**I**) perforin. Individual values for control cells and those with induced FL FoxP3 are shown. (**B**,**D**,**F**,**H**,**J**) Representative flow cytometry plots for control Tregs and those with FL FoxP3. Black histograms in panels (**F**–**J**) indicate non-stimulated cells. Blue and red histograms indicate cells stimulated with the stimulatory cocktail. Black histograms indicate non-stimulated cells *n* = 4. * *p* ≤ 0.05 vs. control cells by Mann–Whitney U test.

The expression of Treg-associated molecules involved in contact-dependent suppressive functioning was investigated by flow cytometry. The proportion of cells expressing CD39 was high in both control Tregs (82.4–95.6%) and Tregs with FL FoxP3 (90.3–97.5%) (Figure 6A,B). The levels of MFI of the suppressive molecules for transfected Tregs are shown in Table 4.

The proportion of cells expressing CD223 was also high and did not differ between control cells (72.1–95.8%) and those with the induced FL splice variant (81.0–96.6%) (Figure 6C,D).

The results of this experiment showed that Tregs with selective expression of FL FoxP3 demonstrated increased suppressive activity. It was associated with the upregulated ability to synthesize granzymes A and B, perforin, and the suppressive cytokines IL-10 and IL-35, as well as with their ability to suppress telomerase. We observed significant induction of the cells with apoptosis-inducing molecules upon FL FoxP3 modulation: 74.3–85.7% of cells expressed granzyme A (Figure 6E,F), and 62.6–76.3% expressed granzyme B (Figure 6G,H), compared to 25.6–46.3% and 14.3–32.2%, respectively, for control Tregs. The proportion of perforin-positive cells was 26.3–46.9 (Figure 6I,J) in control Tregs and increased to 80.0–92.1% after FL FoxP3 induction.

**Figure 7:**
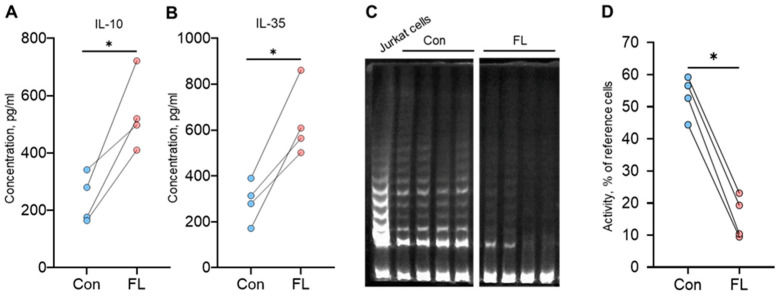
The ability to synthesize suppressive cytokines and to suppress telomerase is elevated in Tregs with full-length (FL) FoxP3. The concentrations of the cytokines (**A**) IL-10 and (**B**) IL-35 in cell culture media were determined by a Bio-Plex assay. (**C**) Telomeric repeat amplification protocol (TRAP) gel electrophoresis used to detect telomerase activity. The Jurkat cell line was used as a reference cell line for telomerase activity measurement. (**D**) The results of TRAP quantification relative to the reference cells. Individual results for control cells (Con) and those with induced FL FoxP3 are shown. *n* = 4. * *p* ≤ 0.05 vs. control cells by Mann–Whitney U test.

The ability of Tregs to produce cytokines involved in contact-independent suppression was examined by measuring their concentrations in cell culture media using the Bio-Plex assay. The concentration of IL-10 was 164.6–341.6 pg/mL in the media from control cells (Figure 7A), while it was significantly increased in the media from FL FoxP3 Tregs at 410.4–721.8 pg/mL. The concentration of IL-35 was also elevated from 171.8 to 390.4 pg/mL for control cells up to 501.8–861.7 pg/mL for FL FoxP3 Tregs (Figure 7B).

The contact-independent suppressive activity of Tregs, attributed to the suppression of telomerase activity in target cells, was measured using the TRAP assay. The residual telomerase activity in target cells co-incubated with control Tregs was 44.4–59.2% of the referenced Jurkat cells (Figure 7C,D). Tregs with the induced expression of the FL splice variant showed a higher ability to suppress telomerase, with residual activity much lower at 9.5–23.1%.

The results of these experiments demonstrated that the increased suppressive activity of Tregs with an induced FL FoxP3 splice variant is associated with the increased synthesis of the pro-apoptotic granzymes A and B and perforin, IL-10, and IL-35 which are responsible for contact-independent suppression and with the increased ability to suppress telomerase in target cells.

## 4. Discussion

The extent of neuroinflammation in ALS patients is associated with a decrease in the number of Tregs and decreased FoxP3 mRNA resulting in disease progression and lower survival chances in ALS murine models and in humans with ALS. Compared to healthy controls, the number of Tregs tends to decline along with the severity of disease progression [15]. The first main finding of our work is the demonstration that the decreased number of Tregs in the peripheral blood of ALS patients and decreased FoxP3 mRNA is associated with a reduced portion of the FL FoxP3 splice variant (Figure 1) and an increased proportion of the truncated ∆2, ∆7, and ∆2∆7 variants in comparison with healthy donors. This observation is also in good agreement with the progression of immunodeficiency, polyendocrinopathy, and enteropathy X-linked syndrome (IPEX syndrome) in patients with truncated FoxP3 splice variants [26].

With regard to the anti-inflammatory action of Tregs, they have been studied as a possible therapeutic option in the regenerative therapy of autoimmune diseases [44,45,46,47,48,49,50] and ALS [51,52,53]. The approaches for the modulation of Tregs’ proliferative and functional activity rely of the use of expansion induced by drugs which exert immunomodulatory effects. Among them, mTOR inhibitor rapamycin [54,55], a nanostructured drug with different oxygen nanobubbles RNS60 [56,57], vitamin D [58,59], and dimethyl fumarate [60,61] are studied the most extensively. Chimeric antigen receptor (CAR) technology has allowed researchers to produce CAR-Tregs with therapeutic relevance towards type 1 diabetes [62], inflammatory bowel disease [63], multiple sclerosis [64], hemophilia [65], vitiligo [66], transplant rejection, and GvHD [67,68]. Genome-editing approaches are applicable to potentiate Treg survival [69,70,71,72], number, and stability during adoptive cell therapy [73,74,75,76,77].

In our work, we demonstrated a fundamentally different approach for the induction of the proliferative and suppressive activity of Tregs by switching of FoxP3 pre-mRNA toward the FL splice variant. For this purpose, we used SSOs which are single-stranded 36-mer synthetic nucleotides that form Watson–Crick base pairs to selectively bind target pre-mRNA [78,79]. Base-pairing to target pre-mRNA blocks the binding of SR proteins to their binding sites, leading to the retention of associated exons and the inclusion of them into mature mRNA. The modulation of cellular functions by SSOs has been shown by us in several previous studies [34,80,81]. Using SSOs targeting sensitive cis-elements on exon 2 and exon 7, we produced Tregs with selective expression of FL FoxP3 (Figure 2). Thus, the second main finding of our study is that SSOs are suitable for the modulation of FoxP3 alternative splicing, directing it to the FL variant.

The most defined immunophenotype of Tregs is CD4^+^CD25^+^CD127^low^. We demonstrated that Tregs with FL FoxP3 have the same profile of these markers as control cells with multiple variants (Figure 3). However, the membrane cytotoxic T-lymphocyte-associated protein receptor CD152 [82] and Ikaros family zinc finger protein Helios [83] were significantly elevated (Figure 3), making them more applicable for the identification of functionally active Tregs [84]. The observation of a high expression of Helios in response to the induction of the FL variant corresponds to the previous studies describing the high stability of FoxP3^+^ Tregs with enhanced Helios [85]. However, in our study we attribute this effect to the primary FL FoxP3′s tight connection with Helios, and our observation is in good accordance to the concern that Helios is just a marker, not a driver, of human Treg stability [86].

The low rate of proliferation is a main limiting factor for producing numbers of Tregs suitable for regenerative purposes [87]. Tregs with FL FoxP3 demonstrated a much higher proliferating rate than control cells (Figure 4). This observation can explain the decreased numbers of Tregs in the peripheral blood of ALS patients shown at Figure 1 and described by Beers at al. [15].

The enhancing of suppressive functional activity of Tregs was also achieved by the induction of FL FoxP3 (Figure 5). Control Tregs with multiple splice variants demonstrated lower activity, confirming reduced Treg suppressive activity in ALS patients [15]. In our study, the induced suppressive activity in FL FoxP3 Tregs was not associated with ectonucleoside triphosphate diphosphohydrolase-1 (CD39), which binds to adenosine receptor A2 on the surface of effector T lymphocytes and suppresses their proliferation by increasing cAMP levels [88], nor with lymphocyte activation gene-3 (CD223), which is responsible for binding to MHC-II molecules of antigen-presenting cells and triggering an inhibitory signaling pathway that prevents the activation of T lymphocytes [89] (Figure 6). The expression of these molecules was the same in control and induced cells. However, the ability to synthesize the serine proteases granzyme A and granzyme B and granzyme-associated perforin, which are responsible for contact-dependent suppressive activity [90], was significantly higher in FL FoxP3 Tregs. Thus, we can suggest that FL FoxP3 is responsible for the implementation of granzyme-mediated suppression of target cells.

Regarding contact-independent suppressive activity, we found enhanced synthesis of the suppressive cytokines IL-10 and IL-35 (Figure 7), as well as the ability to suppress telomerase by Tregs with FL FoxP3.

Two main limitations can be attributed to this study. First, the number of ALS patients and healthy donors is small (*n* = 20) for full statistical validity. However, the data obtained from our groups were sufficient to show the difference in FoxP3 splice variants between ALS and healthy donor groups. Secondly, the antibodies used could not specifically detect the FL FoxP3 protein splice variant. The antibodies used for Western blotting could only recognize exon 2 (clone 150D) and no antibodies against FoxP3 exon 7 have been found to date. In our work, we were only able to show the induction of exon 2 and assumed that this was due to the induction of the FL FoxP3 protein.

## 5. Conclusions

Although the FoxP3 transcription factor is studied as the specific definitive marker of Tregs, several works have suggested that it can also be expressed in human activated non-regulatory CD4^+^ T cells [91,92,93]. Moreover, FoxP3-positive Tregs have homeostatic functions in lymphoid and soft tissues and tumors, making their phenotype highly plastic [94]. We believe that the selective induction of FoxP3 splice variants in different subpopulations of Tregs may become a key to their development, phenotype, and related cellular functions. Of course, the exact molecular links between FoxP3 splice variants and Treg cellular effects remain to be determined. In this paper we have shown that the modulation of FoxP3 alternative splicing towards induction of FL variants by SSOs induces the proliferation and functional suppressive activity of Tregs. We suggest that such a strategy, if further developed, may become a new approach for regenerative support in the treatment of ALS patients.

## Figures and Tables

**Table 1:** Demographics of ALS patients and healthy donors.

Parameter	ALS (*n* = 20)	Healthy Donors (*n* = 20)
Number of female patients	2 (10%)	2 (10%)
Age at study enrollment, mean ± SD *	53.2 ± 12.2	50.6 ± 14.4
Age range	27–70	26–70

* SD—standard deviation (95% confidence interval).

**Table 2:** Clinical data of ALS patients.

Parameter	ALS Group (*n* = 20)
Disease duration (months)	
ALS onset, patients (%)	
spinal onset	17 (85%)
bulbar onset	3 (15%)
ALS stage (King’s staging system)	
Stage 1	-
Stage 2	7 (35%)
Stage 3	10 (50%)
Stage 4	3 (15%)
ALSFRS-R	40.4 ± 3.0 (35–46)

**Table 3:** MFI values of Treg-associated cell markers in control cells and Tregs with induced FL FoxP3. The results are shown as mean ± standard deviation.

Cell Marker	Con	FL
CD4	115.1 ± 4.2	167.4 ± 15.7
CD25	167.9 ± 6.1	180.3 ± 2.8
CD127	40.7 ± 8.9	48.8 ± 3.3
CD152	57.7 ± 9.4	141.7 ± 13.4
Helios	34.5 ± 9.3	111.4 ± 19.9

**Table 4:** MFI values of Treg-associated suppressive molecules in control cells and Tregs with induced FL FoxP3. The results are shown as the mean ± standard deviation.

Suppressive Molecule	Con	FL
CD39	141.2 ± 13.3	153.6 ± 14.2
CD223	130.4 ± 28	143.1 ± 21.8
Granzyme A	54.1 ± 10.0	100.5 ± 6.3
Granzyme B	43.0 ± 10.6	93.1 ± 6.9
Perforin	61.9 ± 8.1	110.6 ± 8.1

## Data Availability

The data presented in this study are available on request from the corresponding author.

## Supplementary Materials

The following supporting information can be downloaded at: https://www.mdpi.com/article/10.3390/biomedicines12051022/s1, Table S1: Manufacturers and catalog numbers for key antibodies, antibody cocktails and reagents used in flow cytometry or Western blotting studies. Figure S1: Individual proportions of FoxP3 mRNA splice variants in Tregs isolated from the peripheral blood of (A) 20 ALS patients and (B) 20 healthy donors. The levels of mRNA were determined by quantitative PCR and normalized to the mean expression of three reference genes: 18S, GAPH, and beta-actin. Patients and donors were ranked according to FL FoxP3splice variant mRNA levels from lowest to highest. Figure S2: Transfection efficiency of Tregs with SSOs. Flow cytometry plots for (A) non-transfected cells; (B) cells transfected with control non-specific oligonucleotides #Con1 & #Con2; and (C) cells transfected with SSOs that specifically inducing the inclusion of exons 2 and 7 #Ins2 & #Ins72. (D,E) The results of transfection efficiency were obtained within 96 h (D,E), and the mean fluorescence intensity (MFI) of the transfected cells showed intracellular loading of SSOs within 96 h (F,G). Results are presented as mean ± standard deviation (*n* = 4). The statistical significance was determined by the Mann–Whitney U test (* *p* ≤ 0.05). Table S2: FoxP3 splice variant mRNA expression was analyzed in Tregs from amyotrophic lateral sclerosis (ALS) patients transfected with SSOs or control oligonucleotides.
