# Supplementary material for: Induction of FoxP3 Pre-mRNA Alternative Splicing to Enhance the Suppressive Activity of Regulatory T Cells from Amyotrophic Lateral Sclerosis Patients
Source: Biomedicines. 2024 May 7;12(5):1022. doi: 10.3390/biomedicines12051022 (PMC11117958; doi:10.3390/biomedicines12051022)
Supplement: Supplementary file 1 [file biomedicines-12-01022-s001.zip › biomedicines-2940268-supplementary.pdf]

Induction of FoxP3 Pre-mRNA Alternative Splicing to Enhance the Suppressive Activity of Regulatory T Cells from Amyotrophic Lateral Sclerosis Patients

Dmitry D. Zhdanov [1,2,*], Yulia A. Gladilina [1], Varvara G. Blinova [1], Anna A. Abramova [1,3], Anastasia N. Shishparenok [1] and Daria D. Eliseeva [3]

[1] Laboratory of Medical Biotechnology, Institute of Biomedical Chemistry, Pogodinskaya St. 10/8, 119121 Moscow, Russia; gladilinaya@ibmc.msk.ru (Y.A.G.); varya.blinova@list.ru (V.G.B.); abramova.neurology@gmail.com (A.A.A.); a.shishparyonok@ibmc.msk.ru (A.N.S.)
[2] Department of Biochemistry, Peoples' Friendship University of Russia named after Patrice Lumumba (RUDN University), Miklukho-Maklaya St. 6, 117198 Moscow, Russia
[3] Research Center of Neurology, Volokolamskoe Shosse, 80, 125367 Moscow, Russia; ddeliseeva@gmail.com
* Correspondence: zhdanovdd@gmail.com or zhdanovdd@ibmc.msk.ru

Abstract: Forkhead box protein 3 (FoxP3) is a key transcription factor responsible for the development, maturation, and function of regulatory T cells (Tregs). The FoxP3 pre-mRNA is subject to alternative splicing, resulting in the translation of multiple splice variants. We have shown that Tregs from patients with amyotrophic lateral sclerosis (ALS) have reduced expression of full-length (FL) FoxP3, while other truncated splice variants are expressed predominantly. A correlation was observed between the reduced number of Tregs in the peripheral blood of ALS patients, reduced total FoxP3 mRNA, and reduced mRNA of its FL splice variant. Induction of FL FoxP3 was achieved using splice-switching oligonucleotides capable of base pairing with FoxP3 pre-mRNA and selectively modulating the inclusion of exons 2 and 7 in the mature mRNA. Selective expression of FL FoxP3 resulted in the induction of $CD127^{low}$, CD152, and Helios-positive cells, while the cell markers CD4 and CD25 were not altered. Such Tregs had an increased proliferative activity and a higher frequency of cell divisions per day. The increased suppressive activity of Tregs with the induced FL FoxP3 splice variant was associated with the increased synthesis of the pro-apoptotic granzymes A and B, and perforin, IL-10, and IL-35, which are responsible for contact-independent suppression, and with the increased ability to suppress telomerase in target cells. The upregulation of Treg suppressive and proliferative activity using splice-switching oligonucleotides to induce the predominant expression of the FoxP3 FL variant is a promising approach for regenerative cell therapy in Treg-associated diseases.

Keywords: regulatory T cells; alternative splicing; FoxP3; suppressive activity; splicing-switching oligonucleotides; amyotrophic lateral sclerosis

Table S1. Manufacturers and catalog numbers for key antibodies, antibody cocktails and reagents used in flow cytometry or Western blotting studies.

	Name	Manufacturer	Catalog number
1	Treg Surface Marker Analysis Cocktail	Miltenyi Biotec, Bergisch Gladbach, Germany	#130-096-082
2	Anti-CD4–FITC		#130-113-775
3	Anti-CD25–APC		#130-123-832
4	Anti-CD127–FITC		#130-113-409
5	Anti-CD152–APC		#130-123-812
6	Anti-CD39–FITC		#130-125-094
7	Anti-Helios-PE		#130-104-033
8	Anti-Granzyme A-PE		#130-123-973
9	Anti-Granzyme B-PE		#130-116-654
10	Anti-Perforin-FITC		#130-096-668
11	Transcription Factor Buffer Set	BD Pharmingen, East Rutherford, NJ	#562574
12	Stimulation Cocktail + Protein Transport Inhibitor	eBioscience Inc., San Diego, CA	#00-4975-03
13	Protein transport inhibitor		#00-4980-93,
14	Anti-mouse/ rat/human FoxP3, clone 150D	BioLegend, San Diego, CA	#320001
15	Anti-human FoxP3, clone 259D		#320201
16	Anti-GAPDH, clone 6C5	Abcam, Cambridge, MA	#ab8245

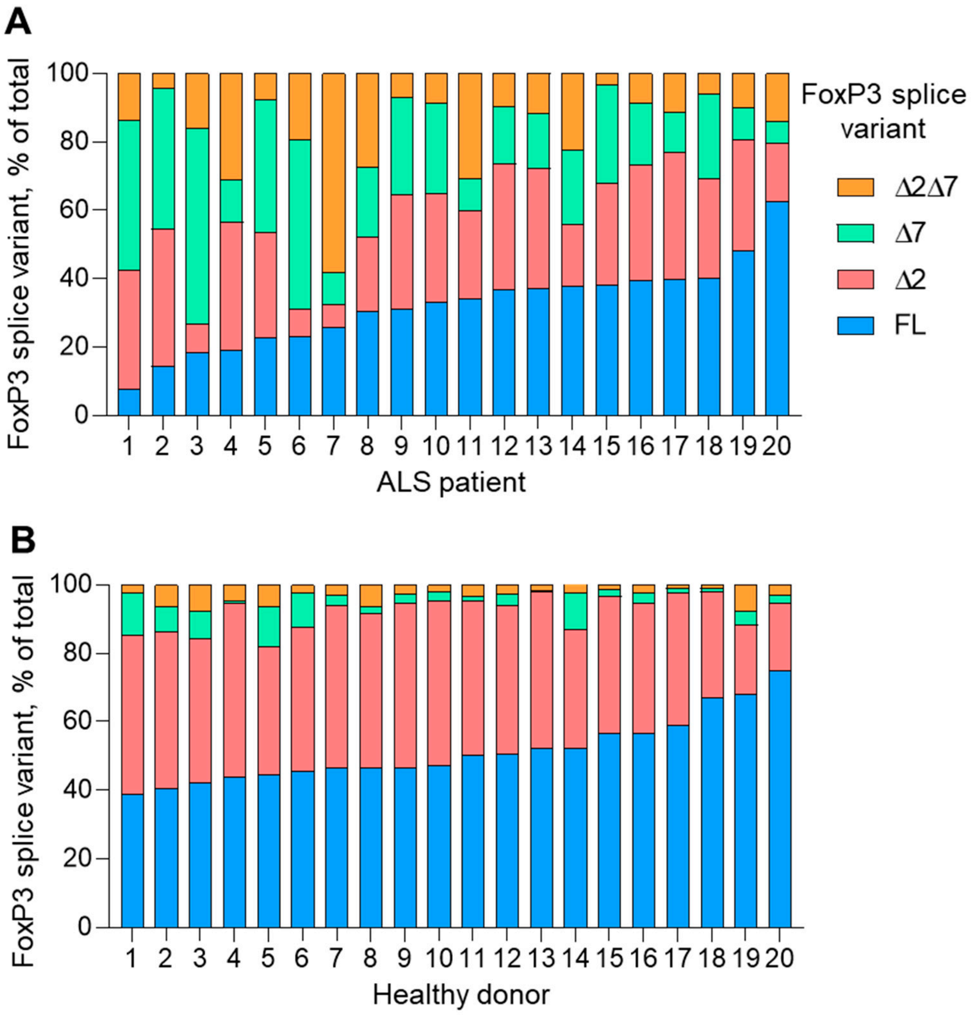

Figure S1. Individual proportions of FoxP3 mRNA splice variants in Tregs isolated from the peripheral blood of (A) 20 ALS patients and (B) 20 healthy donors. The levels of mRNA were determined by quantitative PCR and normalized to the mean expression of three reference genes: 18S, GAPH, and beta-actin. Patients and donors were ranked according to FL FoxP3splice variant mRNA levels from lowest to highest.

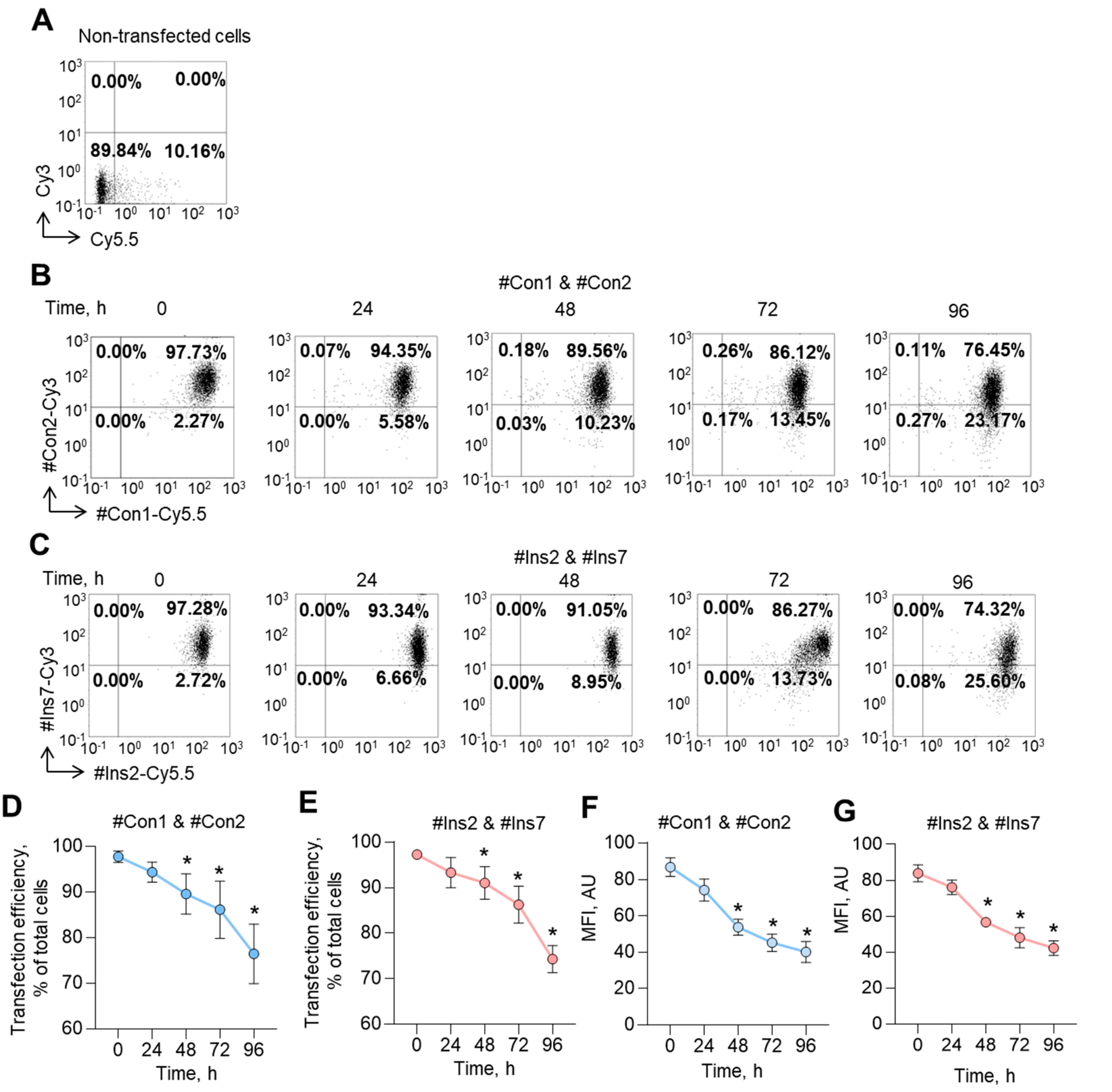

Figure S2. Transfection efficiency of Tregs with SSOs. Flow cytometry plots for (A) non-transfected cells; (B) cells transfected with control non-specific oligonucleotides #Con1 & #Con2; and (C) cells transfected with SSOs that specifically inducing the inclusion of exons 2 and 7 #Ins2 & #Ins72. (D,E) The results of transfection efficiency were obtained within 96 hours (D,E), and the mean fluorescence intensity (MFI) of the transfected cells showed intracellular loading of SSOs within 96 hours (F,G). Results are presented as mean ± standard deviation (n=4). The statistical significance was determined by the Mann–Whitney U test (* $p \leq 0.05$).

Table S2. FoxP3 splice variant mRNA expression was analyzed in Tregs from amyotrophic lateral sclerosis (ALS) patients transfected with SSOs or control oligonucleotides.

Transfection	FL variant	Δ2 variant	Δ7 variant	Δ2Δ7 variant
	**ALS patient 1**			
#Ins2 & #Ins7	0.379	0.001	0.011	0.006
#Con1 & #Con2	0.152	0.123	0.042	0.028
	**ALS patient 2**			
#Ins2 & #Ins7	0.328	0.006	0.003	0.008
#Con1 & #Con2	0.139	0.1153	0.068	0.030
	**ALS patient 3**			
#Ins2 & #Ins7	0.297	0.005	0.002	0.004
#Con1 & #Con2	0.122	0.106	0.119	0.034
	**ALS patient 4**			
#Ins2 & #Ins7	0.275	0.004	0.006	0.002
#Con1 & #Con2	0.033	0.114	0.115	0.035

Tregs were transfected with SSOs that could induce the insertion of exons 2 and 7 (i.e., #Ins2 & #Ins7 oligonucleotides) or control nonspecific oligonucleotides #Con1 & #Con2. After 96 hours, qPCR was performed and the levels of investigated mRNAs were normalized to the average expression of three reference genes: 18S, GAPDH, and beta-actin. FL, full-length splice variant; Δ2, splice variant with deleted exon 2; Δ7, splice variant with deleted exon 7; Δ2Δ7, splice variant with deleted both exon 2 and exon 7.
